# George M. Beard, A.M., M.D.

**Published:** 1883-02

**Authors:** 


### Obituary.

It is with feelings of deep regret that we chronicle the death of Dr George Miller Beard, of this city. The doctor had been suffering from an alveolar abscess for some days, when, suddenly and unexpectedly pleuro-pneumonia set in, terminating fatally on January 23.

Dr. Beard was a distinguished practitioner and an untiring writer. He has often been heard through the columns of the INDEPENDENE PRACTITIONER, his contributions being of the highest interest, from their practical bearing and peculiar and original scope. He had also been known as a lecturer. Among the principal events of the doctor's busy life was his controversy with the Rev. Dr. Theodore L. Cuyler, of Brooklyn, on the subject of temperance; his public contest with the faculty of Yale College, many of whom had been experimenting with Brown, the mind reader, which at the time created great excitement.

Dr. Beard was connected professionally as an expert with the trials of Cadet Whittaker and Guiteau. In the case of the former, he testified for the defence, taking the position that the accusation against Whittaker was unjust. In the case of Guiteau, Dr. Beard's position was that the assassin was insane. He predicted, in detail, the manner in which Guiteau would meet his fate, with insane speech on his lips, and this prediction was fulfilled.

Dr. Beard's investigations into sea sickness were of great value, and the plan of treatment has now been successfully carried out on every sea, for the longest voyages. At the end of last year, and even within the past few weeks, Dr. Beard continued his muscle reading studies with Stuart Cumberland, the English mind reader. With the inventor Edison he spent much time in experimenting. Dr. Beard's writings and researches have had more influence in Germany than any other country, and many of his works have been translated into German.

Dr. Beard was born at Montville, Conn., May 8, 1839. He entered the Academical Department of Yale College, in 1858, and graduated four years later. In college, he was a prominent scholar and writer, receiving a prize for English composition and the Townsend premium. He was also editor of the *Yale Literary Magazine.* After graduating in arts, he studied one year in the Medical Department of Yale College, and from 1863 to 1864, was Acting Assistant Surgeon in the United States Navy. In 1866, he graduated at the College of Physicians and Surgeons in this city.

Just before his death, he frequently repeated, "I wish it were possible for me to record, for the sake of science, the thoughts of a dying man. This final battle that I am going through with would be interesting." Indeed, these words were almost the last he uttered. Thus he passed away, with the key to his whole life upon his lips.

